# YTHDF2 in inflammation: Mechanisms and therapeutic strategies

**DOI:** 10.1016/j.gendis.2025.101909

**Published:** 2025-10-28

**Authors:** Junxiu Liu, Senxu Lu, Chuanhuai Chen, Xiaobo Lin, Lijuan Xia, Pansheng Xu, Jinjin Shao, Luxi Yang, Wenhai Huang, Lijiang Zhang

**Affiliations:** a Zhejiang Provincial Key Laboratory of Drug Discovery and Safety Evaluation for Inflammatory Chronic Diseases, Center of Safety Evaluation and Research, Hangzhou Medical College, Hangzhou, Zhejiang 310053, China; b Department of Orthopedic Surgery, The Second Affiliated Hospital, Zhejiang University School of Medicine, Hangzhou, Zhejiang 310009, China; c Orthopedics Research Institute of Zhejiang University, Hangzhou, Zhejiang 310058, China; d Key Laboratory of Motor System Disease Research and Precision Therapy of Zhejiang Province, Hangzhou, Zhejiang 310058, China; e Zhejiang Key Laboratory of High-level Biosafety and Biomedical Transformation, Hangzhou Medical College, Hangzhou, Zhejiang 311305, China

**Keywords:** Inflammation, N6-methyladenosine, RNA modification, Therapeutic drugs, YTHDF2

## Abstract

Inflammation is a double-edged sword in biology. Moderate immune responses effectively eliminate pathogens and promote tissue repair, while excessive or persistent inflammation drives acute and chronic diseases. N6-methyladenosine (m^6^A), a central RNA epigenetic modification, dynamically regulates inflammatory initiation, amplification, and resolution. Among m^6^A-binding proteins, YTHDF2—the first identified mammalian m^6^A reader—modulates inflammatory responses by recognizing m^6^A/m^5^C sites to control RNA stability and translation. This review reports novel insights into the role of YTHDF2 in regulating immune cell functions and inflammatory signaling pathways across various disease contexts. Specifically, it systematically summarizes the molecular mechanisms through which YTHDF2 contributes to the pathogenesis of inflammatory diseases and reviews recent advances in the development of selective therapeutic agents targeting YTHDF2. Additionally, the functional complexity of YTHDF2 within specific pathological environments is discussed, and the current challenges facing the translation of these findings into targeted therapies are outlined. This review is expected to serve as a theoretical foundation for prospective therapeutic strategies employing novel epigenomic regulation-based approaches to treat inflammatory diseases.

## Introduction

Inflammation is a defense mechanism activated by the host immune system against harmful stimuli like infection, injury, or stress. Insufficient inflammatory responses may result in persistent pathogen infections and impaired tissue repair. Conversely, excessive or unresolved inflammation can drive chronic/systemic inflammatory diseases, including tumors, neurodegenerative disorders, hepatitis, nephritis, and myocarditis.^1^, ^2^, ^3^, ^4^, ^5^ Thus, modulating inflammatory responses is crucial for maintaining physiological homeostasis.

A typical inflammatory response involves four key components: inducers, sensors, mediators, and effectors.^6^ Inducers comprise both exogenous stimulants, including bacterial lipopolysaccharides, viruses, foreign substances, and toxic compounds (pathogen-associated molecular patterns, PAMPs), and endogenous stimulants, such as dead cells, damaged tissues, and their released damage-associated molecular patterns (DAMPs).^7^ When inducers are recognized by inflammatory receptors such as Toll-like receptors (TLRs), NOD-like receptors (NLRs), RIG-I like receptors (RLRs), C-type lectin receptors, or AIM2-like receptors (ALRs), they activate inflammatory signaling pathways or inflammasomes.^8^ This triggers the production of inflammatory mediators, including proinflammatory cytokines (TNF, IL1β, IL6), chemokines, complement proteins, and coagulation factors. These mediators recruit immune effector cells (macrophages, neutrophils, dendritic cells, and natural killer (NK) cells) to target tissues, amplifying immune responses. Simultaneously, they regulate non-immune cells (epithelial cells, endothelial cells, and fibroblasts) to promote tissue repair.^9^ Thus, at the molecular or cellular level, inflammatory response regulation primarily involves inflammatory factors, signaling pathways, and immune effector cell activity.

**Figure 1:**
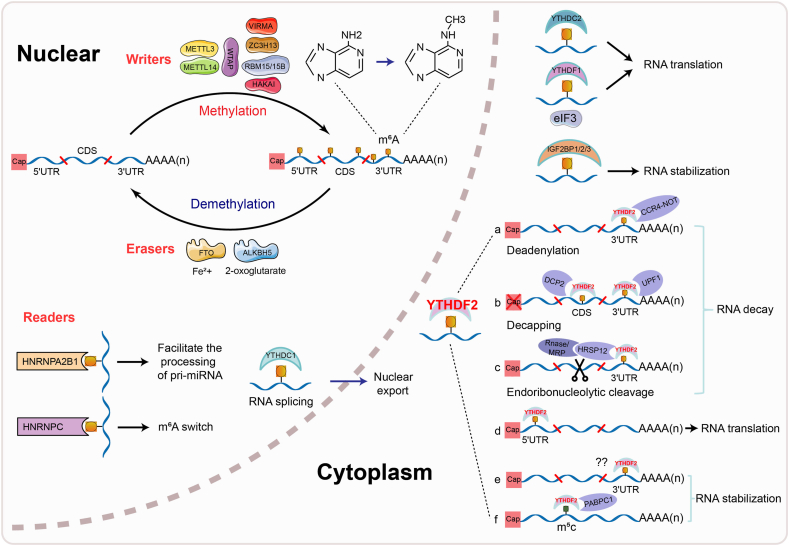
Mechanisms of m^6^A methylation modification with its core reader YTHDF2. m^6^A methylation orchestrates RNA processing, transport, stability, and translation through writers, erasers, and readers. As a core m^6^A reader, YTHDF2 primarily drives RNA degradation via three interconnected pathways: deadenylation, decapping, and exdoribonuleolytic cleavage upon recognizing m^6^A sites in the 3′UTR. It also binds to m^6^A in the CDS region, recruiting DCP2 to translocate mRNA to processing bodies (P-bodies) for accelerated decay through decapping. Conversely, YTHDF2 enhances protein translation when bound to m^6^A in the 5′UTR and stabilizes RNA by recognizing m^6^A/m^5^C sites in the 3′UTR.

Promoting the precise regulation of inflammatory responses is essential for preventing disease progression. Inflammation is influenced by genetic factors and various epigenetic mechanisms, such as m6A RNA methylation, DNA methylation, histone modifications, and non-coding RNA-mediated regulation. These epigenetic processes dynamically modulate chromatin structure and the expression of immune-related genes without changing the DNA sequence, thereby influencing the intensity and duration of inflammatory responses. Studies have shown that N6-methyladenosine (m^6^A), the predominant RNA epigenetic modification in eukaryotic transcripts, regulates inflammatory disease progression by controlling inflammatory factor expression, disrupting signaling pathways, and dysregulating immune cell activation through RNA metabolism.^10^, ^11^, ^12^ m^6^A methylation is a dynamic, reversible epigenetic process that selectively regulates RNA processing, transport, stability, and translation efficiency through coordinated actions of methyltransferases (writers), demethylases (erasers), and m^6^A-binding proteins (readers) (Fig. 1).^13^, ^14^, ^15^ The METTL3/METTL14/WTAP methyltransferase complex (MTC) recognizes the DRACH motif of RNA and preferentially methylates mRNA coding regions (CDSs), 3′UTRs, and stop codon proximity. Cofactors, including VIRMA, RBM15/RBM15B, ZC3H13, and HAKAI, facilitate MTC recruitment for site-specific m^6^A deposition.^16^, ^17^, ^18^ m^6^A demethylation is primarily catalyzed by FTO and ALKBH5, which utilize Fe^2+^ and α-ketoglutarate (α-KG) as cofactors.^19^ m^6^A reader proteins determine the fate of m^6^A-modified RNAs. These comprise three families: YTH domain proteins (YTHDF1-3, YTHDC1/2), hnRNPs (HNRNPA2B1, HNRNPC/G), and IGF2BPs (IGF2BP1-3). All recognize m^6^A sites to regulate RNA metabolism.^13^ For example, YTHDF1 binds eIF3 to initiate mRNA translation^20^; YTHDF3 promotes translation via YTHDF1 but induces degradation via YTHDF2^21^; YTHDC1 regulates mRNA splicing and nuclear export^22^^,^^23^; and YTHDC2 enhances target mRNA translation while reducing its cellular abundance.^24^ Moreover, the hnRNP and IGF2BP families regulate RNA splicing, degradation, stability, and translation through m^6^A recognition.^25^^,^^26^

Among the m^6^A reader proteins identified in mammals, YTHDF2 plays a distinctive role in modulating inflammatory responses. Within the cytoplasmic YTHDF family, YTHDF1 exhibits a relatively straightforward regulatory mechanism compared to YTHDF2, primarily influencing inflammation by modulating the translation efficiency of target mRNAs. For example, YTHDF1 promotes intestinal inflammation by enhancing the translation of XPO1.^27^ In contrast, YTHDF3 does not directly regulate target genes but rather cooperates with other proteins to exert its effects. A previous report demonstrated that YTHDF3 collaborates with METTL3 to accelerate the degradation of PTX3—a key regulator involved in inflammation and asthma—and contributes to M2 macrophage activation, thereby modulating inflammatory responses.^28^ Compared to YTHDF1 and YTHDF3, YTHDF2 exhibits multi-layered and dynamic regulatory control over target RNAs in inflammation. It plays a central role in the pathogenesis of various diseases and malignancies, including tumors,^29^ cardiovascular disorders,^30^ bone and joint diseases,^31^ and central nervous system conditions,^32^ by regulating RNA stability, degradation, and translation. More importantly, it directly targets inflammatory molecules and pathways, in turn exerting a profound influence on the inflammatory signaling cascade.

Although several reviews have discussed the roles of YTHDF2 in cancers^33^^,^^34^ and neurological diseases,^35^ a comprehensive evaluation focusing on its systematic functions in inflammatory responses is lacking. This is particularly evident in areas such as the inflammatory microenvironment, crosstalk among inflammatory pathways, disease-specific mechanisms, and targeted therapeutic strategies.

This review highlights the central role of YTHDF2 in the initiation, amplification, and resolution of inflammation through epitranscriptomic regulation. It summarizes the functional diversity and mechanistic novelty of YTHDF2 across various immune cell types and key inflammatory signaling pathways, and systematically reviews recent advances in the development of agonists and inhibitors targeting YTHDF2. Furthermore, we propose therapeutic intervention strategies focused on YTHDF2, aiming to offer new perspectives and a strategic foundation for the development of precise epitranscriptomic therapies for inflammatory diseases.

## Mechanism of YTHDF2 in RNA regulation

As a core member of the YTH family, the primary function of YTHDF2 is to regulate gene expression by degrading its target mRNAs. Primarily cytoplasmic, it recognizes m^6^A sites in 3′UTRs and mediates mRNA degradation through three pathways: deadenylation via CCR4-NOT recruitment; decapping through UPF1 interaction; and exonucleolytic digestion by recruiting HRSP12 to RNase P/MRP complexes (Fig. 1).^36^ YTHDF2 also recognizes m^6^A sites in coding regions (CDSs) and recruits DCP2 to mediate mRNA transfer to P-bodies for decapping-mediated decay.^37^

YTHDF2 also promotes protein translation, and enhances RNA stability. Studies have reported that YTHDF2 can bind to m^6^A sites in the 5′ UTR region to promote protein translation. For example, it forms a complex with the translation initiation factor eIF3F and the RNA helicase DDX1, binds to m^6^A-modified tubulin CKAP2 mRNA, and directly enhances its translation efficiency without affecting its RNA half-life, thereby contributing to tumor drug resistance.^38^ Additionally, YTHDF2 can stabilize RNA by recognizing m^6^A sites located in the 3′ UTR or 5′ UTR of mRNAs. Elevated O-GlcNAcylation of YTHDF2 has been shown to increase the 3′ UTR activity of cyclin MCM2/5 transcripts in a m^6^A-dependent manner, stabilizing MCM2/5 mRNA, promoting cell cycle progression, and exacerbating HBV-induced hepatocellular carcinogenesis.^39^ Similarly, YTHDF2 binds to the m^6^A site in the 3′ UTR of ACER2 mRNA to stabilize it, thereby activating the PI3K/AKT and ERK pathways and promoting tumor progression.^40^ YTHDF2 can also promote m^6^A modification in the 5′ UTR of the transcription factor OCT4 mRNA, which enhances OCT4 expression and facilitates the phenotype of liver cancer stem cells and cancer metastasis.^41^

Notably, YTHDF2 can also stabilize mRNA expression by binding to m5C modifications on mRNAs, and—in coordination with its canonical mRNA degradation function—promote tumor immune escape.^42^ YTHDF2 stabilizes mRNAs encoding ATP synthase subunits by recognizing their m5C sites and recruiting poly(A)-binding protein C1 (PABPC1), thereby enhancing ATP synthesis efficiency and supplying energy for rapidly proliferating cancer cells. On the contrary, YTHDF2 recognizes m^6^A-modified CD19 and MHC class II mRNAs, promotes their degradation, reduces the visibility of cancer cells to the immune system, and consequently helps tumors to evade immune-mediated killing.^42^

## YTHDF2 regulation of inflammation-related immune cell function

YTHDF2 is highly expressed in various cancers, including liver cancer, colorectal cancer, and gliomas, and its role in promoting tumorigenesis has been extensively documented.^34^ The tumor microenvironment is characterized by chronic inflammation and numerous inflammatory factors, chemokines, and dysfunctional immune cells.^43^ Growing evidence suggests that the oncogenic function of YTHDF2 largely arises from its significant regulatory role in the tumor immune microenvironment—a mechanism consistent with its actions in classical inflammatory responses.

Acting as an “immunometabolic checkpoint”, YTHDF2 influences cancer progression owing to its roles in both stabilizing and degrading RNA. Its tumor-promoting mechanisms can be summarized in four aspects. First, YTHDF2 directly suppresses antitumor immunity by degrading key mRNA molecules, thereby impairing the cytotoxic capacity of immune cells.^42^^,^^44^ Second, it facilitates the recruitment of immunosuppressive cells by modulating chemokine secretion from tumor cells, thereby fostering an immunosuppressive microenvironment that supports tumor growth.^45^, ^46^, ^47^ Third, YTHDF2 sustains the activation of pro-tumor and proinflammatory signaling pathways, such as NF-κB and PI3K-AKT, through the degradation of negative regulators, thus further driving malignant progression.^40^^,^^48^ Finally, it promotes tumor cell survival under stress conditions by inhibiting cell death mechanisms such as ferroptosis and pyroptosis.^49^^,^^50^ Notably, when YTHDF2 is highly expressed in NK cells, it enhances their cytotoxic activity and suppresses tumor growth.^51^, ^52^, ^53^

**Figure 2:**
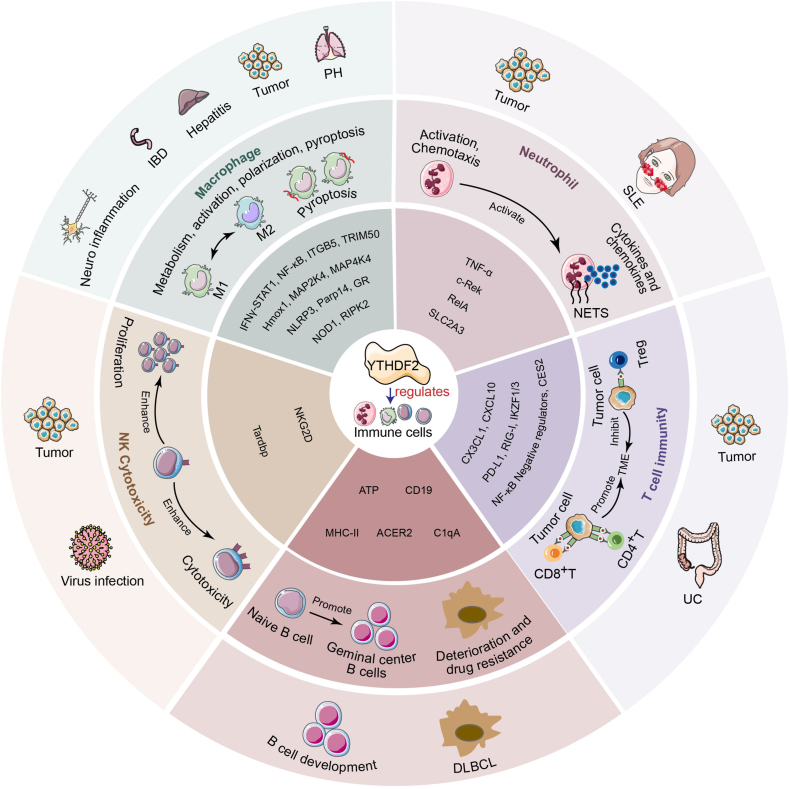
YTHDF2 governs inflammatory immune cell behavior. YTHDF2 regulates downstream target RNA expression via m^6^A epigenetics and thus modulates macrophage functions (metabolism, activation, polarization, and pyroptosis), neutrophil activation/chemotaxis, NK cytotoxicity, and T/B-cell immunity to accelerate disease progression.

Overall, the role of YTHDF2 in various cancers represents a convergence of its immune and inflammatory regulatory functions within the tumor microenvironment. The functions of YTHDF2 influence transcriptional regulation in tumor cells with immune cell functionality, creating and maintaining an immunosuppressive inflammatory niche that supports tumor proliferation, invasion, metastasis, and immune evasion. Therefore, investigating the YTHDF2-dependent m^6^A regulatory mechanisms in inflammation-related immune cells across inflammatory diseases and cancers is essential for developing precise targeted therapeutic strategies (Fig. 2).

## The role of YTHDF2 in macrophage activation, polarization, and pyroptosis

Macrophages serve as central regulators of inflammatory responses. In the early stages of embryonic development, they are derived primarily from yolk sac-derived myeloid progenitors. These precursors populate specific tissues prenatally (e.g., microglia in the brain and Kupffer cells in the liver) and maintain homeostasis via local self-renewal. Postnatally, most macrophages originate from bone marrow hematopoietic stem/progenitor cell (HSPC)-derived monocytes. These monocytes migrate to peripheral tissues through the blood circulation and differentiate into macrophages under the induction of specific cytokines (such as M-CSF and GM-CSF), participating in the process of inflammation or injury repair.^54^^,^^55^

As key immune effectors, macrophages regulate inflammatory responses through distinct polarization states. Upon pathogen invasion, they transition from M0 resting macrophages to proinflammatory M1 macrophages, which combat infection by releasing inflammatory mediators (IL1β, IL6, and TNFα) and reactive oxygen species (ROS) to enhance pathogen clearance. Conversely, during tissue repair, anti-inflammatory M2 macrophages secrete immunoregulatory cytokines (IL4, IL10, and TGFβ) to suppress inflammation and promote tissue regeneration. However, the imbalance of macrophage polarization is the pathological basis driving many inflammatory diseases. In chronic inflammatory diseases such as diabetic retinopathy, excessive M1 polarization exacerbates vascular dysfunction and inflammatory progression.^56^ Conversely, in the tumor immune microenvironment, M2-polarized tumor-associated macrophages (TAMs) suppress T-cell-mediated immunity, facilitating tumor immune evasion.^57^

Emerging evidence has demonstrated that YTHDF2 regulates macrophage activation, polarization, and pyroptosis through epigenetic modifications of RNA and exert varied functions in numerous disease processes (Table 1). Specifically, YTHDF2 modulates the tumor immune microenvironment by controlling macrophage activation. Under YTHDF2-deficient conditions, it mediates TAM metabolic reprogramming by targeting the IFNγ-STAT1 pathway^58^ and recruits TAM to tumor cells through CX3CL1 chemotaxis.^59^ YTHDF2 deficiency also inhibits the ubiquitination of PGK1 by increasing the expression of TRIM50, thereby suppressing the glycolytic metabolism of tumor cells.^60^ It also enhances CD8^+^ T-cell-mediated tumor immunity and inhibits tumor growth by promoting the expression of antitumor markers (IL1β, IL6, and TNFα) in macrophages.^61^ Furthermore, in pulmonary hypertension, YTHDF2 promotes alveolar macrophage activation by accelerating Hmox1 mRNA degradation, which exacerbates disease progression.^62^^,^^63^

**Table 1:** The role of YTHDF2 in inflammation-related immune cells.

Cell type	Disease	Expression	Molecular mechanisms	Function	Ref
TAMs	Tumor	Deficiency	Targeting the IFNγ-STAT1 pathway and mediate the metabolic reprogramming of TAM	Enhancing antitumor immunity	58
	Gastric cancer	Up-regulated	Decreasing TRIM50, thereby enhancing the glycolytic metabolism	Promoting cancer	60
	ICC	Up-regulated	Reducing the level of PD-L1 protein on the surface of tumor cells thereby decreasing the recruitment of TAMs	Promoting immune evasion	64
RAW264.7	Tumor	Deficiency	Enhancing CX3CL1, thereby recruiting macrophages and damaging the glycolytic metabolism	Enhancing antitumor immunity	59
	IBD	Down-regulated	Enhancing ITGA5 to promote inflammation	Promoting IBD	65
BMDMs	Breast cancer	Deficiency	Enhancing IL1β, IL6, and TNFα	Inhibiting cancer	61
AMs	PH	Up-regulated	Decreasing Hmox1 to accelerate the activation of macrophages and promote the proliferation of PASMCs	Promoting PH	62,63
THP-1	Diabetic Vasculopathy	Up-regulated	Decreasing FGF2 to inhibit PI3K-AKT pathway thereby promoting M1 macrophages polarization	Promoting diabetic Vasculopathy	66
BMDMs	UC	Up-regulated	Cooperate with WTAP to reduce CES2 thereby promoting M1 macrophages polarization and recruiting CD4^+^ T cell	Promoting UC	67
CD4^+^ T cell					
Microglia	Neuro-inflammation	Up-regulated	Decreasing Parp14 thereby leading to the imbalance of microglial homeostasis	Promoting neuro-inflammation	68
Neutrophil	SLE	Down-regulated	Enhancing TNFα to promote inflammation	Promoting SLE	69
	LUAD	Up-regulated	NETs increase YTHDF2 in LLC thereby reducing SLC2A3 to inhibit ferroptosis by inhibiting CD8^+^ T cell activity	Promoting LUAD	49
TANs	PTC	/	Cooperate with low-expressed METTL3 to enhance c-Rek and RelA to elicit IL-8 thereby increasing the recruitment of TANs	Promoting PTC	70
NK	Tumor and virus infection	Deficiency	Failing to form a positive feedback loop with STAT5 or degrade Tardbp, thereby suppressing NK proliferation and survival	Down-regulating the antitumor and anti-viral activities	51
	CRC	Down-regulated	Decreasing NKG2D thereby inhibiting the cytotoxic efficacy of NK	Promoting CRC	52
CD8^+^ T cell	Liver cancer	Down-regulated	Decreasing Cx3cl1 to inhibit CD8^+^ T cell recruitment and cytotoxic efficacy	Promoting liver cancer	45
	CRC	Down-regulated	Enhancing CXCL10 thereby increasing the recruitment of CD8^+^ T cell	Inhibiting CRC	46
	BLCA	Up-regulated	Decreasing DDX58 thereby increasing the recruitment of CD8^+^ T cell	Promoting BLCA	44
	ICC	/	Cooperate with low-expressed ALKBH5 to decrease PD-L1 thereby increasing CD8^+^ T cell proliferation and cytotoxic efficacy	Inhibiting ICC	64
	Tumor	Deficiency	Leading to mitochondrial dysfunction and chromatin inactivation of CD8^+^ T cell by increasing IKZF1/3	Inhibiting tumor immunity	71
Treg	Tumor	Deficiency	Increasing NF-κB negative feedback regulator thereby relieving TME suppression induced by Treg	Inhibiting tumor	47
B Cell	B Cell malignancies	Up-regulated	Enhancing ATP synthesis by recognizing m5C sites and recruiting PABPC1, while also degrading m6A-modified CD19 and MHCⅡ mRNAs	Promoting B cell malignancies	42
	DLBCL	Up-regulated	Enhancing ACER2 to promote ceramide catabolism, thereby activating PI3K/AKT and ERK pathways	Promoting DLBCL	40
		Up-regulated	Decreasing C1qA thereby making rituximab resistance in DLBCL	Promoting DLBCL	72

In addition to regulating macrophage activation, YTHDF2 critically regulates both M1/M2 polarization dynamics and inflammatory responses. The increased expression of YTHDF2 promotes the polarization of macrophages toward M1^66^, ^67^, ^68^^,^^73^ and cooperates with METTL3 to activate the NLRP3 inflammasome and NF-κB inflammatory pathway, intensifying the inflammatory response process.^74^, ^75^, ^76^ Following lipopolysaccharide (LPS) stimulation, tissue-resident macrophages—particularly microglia in the central nervous system and Kupffer cells in the liver—exhibit up-regulated YTHDF2 expression. This elevation exacerbates neuroinflammation and hepatitis progression through the enhanced degradation of Parp14^68^ and glucocorticoid receptor mRNAs.^77^ When YTHDF2 was silenced, the mRNA stability of MAP2K4, MAP4K4,^78^ NOD1, and RIPK2^79^ increased, which led to the hyperactivation of the mitogen-activated protein kinase (MAPK), NF-κB, and NOD1 pathways and subsequently intensified inflammatory responses in LPS-stimulated macrophages. Furthermore, in enterotoxigenic *Bacteroides fragilis* (ETBF)-induced inflammatory bowel disease, reduced YTHDF2 expression impairs its cooperative function with METTL3 to degrade ITGB5 mRNA.^65^ The consequent ITGB5 up-regulation promotes the secretion of macrophage-derived inflammatory factors, thus exacerbating disease progression.^65^ These findings demonstrate that YTHDF2’s regulation of macrophage polarization and inflammatory responses critically depends on the pro- or anti-inflammatory nature of its downstream targets.

Furthermore, YTHDF2 can decrease the stability of NLRP3 mRNA in a rat model induced by homocysteine (Hcy) and alleviate microglial pyroptosis by inhibiting the NLRP3/caspase-1/GSDMD pathway.^80^

In summary, YTHDF2 can dynamically regulate multiple functional states of macrophages through recognition of m^6^A modifications, thus playing a key role in inflammatory responses, polarization balance, and cell death. Its activity is highly context-dependent, exhibiting dual regulatory roles that either promote or suppress inflammation depending on the disease state and specific stimulatory conditions. However, the mechanisms through which YTHDF2 operates across different macrophage subtypes in various inflammatory diseases remain poorly understood. Future studies should focus on elucidating the precise molecular networks by which YTHDF2 modulates macrophage function under diverse pathological conditions and explore its potential as a therapeutic target. Such efforts could elucidate new strategies for the precise treatment of inflammation-related diseases.

## The role of YTHDF2 in neutrophil activation and chemotaxis

Neutrophils are the earliest innate immune cells recruited to sites of infection and inflammation, playing a key role throughout the initiation, amplification, and resolution phases of inflammatory responses. Following tissue damage or pathogen invasion, local endothelial cells and macrophages release mediators, including chemokines such as IL8, CXCL1, and CXCL2, the complement fragment C5a, and leukotriene B4 (LTB4). These mediators guide neutrophils to migrate from the vasculature to the inflammatory site via interactions with adhesion proteins such as P-selectin, E-selectin, and integrin ligands. Subsequently, activated neutrophils contribute to regulating inflammation through phagocytosis, degranulation, the release of various mediators, and the formation of neutrophil extracellular traps.^81^^,^^82^

YTHDF2 regulates neutrophil activation and chemotaxis, playing a significant role in cancers^49^^,^^70^^,^^83^ and autoimmune diseases^69^ (Table 1). In patients with systemic lupus erythematosus, YTHDF2 expression is reduced, while the inflammatory factor TNFα is markedly elevated. When neutrophils from healthy individuals are cultured in SLE patient serum under specific conditions, they exhibit patterns of YTHDF2 down-regulation and TNFα up-regulation identical to those observed in neutrophils under this disease condition. This suggests that decreased YTHDF2 expression impairs the degradation of TNFα mRNA, thereby amplifying inflammatory responses and accelerating systemic lupus erythematosus progression.^69^

Furthermore, YTHDF2 influences the chemotaxis of tumor-associated neutrophils (TANs) by modulating chemokine secretion in tumor cells.^70^ In thyroid cancer cells, significantly reduced METTL3 expression impairs the ability of YTHDF2 to recognize m^6^A sites on c-Rel and RelA mRNAs. This results in elevated c-Rel and RelA expression, which activates the NF-κB pathway and enhances IL8 secretion from thyroid cancer cells. Consequently, substantial TAN recruitment to tumor sites occurs, accelerating tumor progression.^70^

Notably, NEFs released by neutrophils can, in turn, regulate tumor immunity by modulating YTHDF2 levels in tumor cells.^49^ Phorbol 12-myristate 13-acetate (PMA)-stimulated neutrophils release NETs, which subsequently mediate SLC2A3 mRNA degradation through increased YTHDF2 expression in lung adenocarcinoma LLC cells. This reduced SLC2A3 expression inhibits ferroptosis in tumor cells and suppresses CD8^+^ T-cell activity, which ultimately promotes tumor immune escape.^49^

In summary, YTHDF2 amplifies inflammation by enhancing neutrophil proinflammatory secretion while disrupting the tumor immune microenvironment through chemokine regulation in tumor cells and neutrophil migration control. Conversely, neutrophil extracellular traps promote tumor immune escape by modulating tumor cell death pathways. However, when neutrophils are recruited in large quantities into tumor cells, how YTHDF2 influences tumor progression via N1 (antitumor)/N2 (pro-tumor) neutrophil polarization remains unknown. This critical gap warrants further investigation.

## The role of YTHDF2 in NK cytotoxicity

NK cells serve as the primary effector cells of innate immunity, rapidly eliminating abnormal targets (including virus-infected, cancerous, and stressed cells) through antigen-independent cytotoxicity. Their killing mechanisms operate through two principal pathways: 1) release of perforin/granzyme particles and cytokines (IFNγ, TNFα) that induce target cell apoptosis,^84^^,^^85^ and 2) engagement of activating surface receptors (CD16, NCRs [NKp30/40/44/46], NKG2D).^86^^,^^87^ Conversely, inhibitory receptors (NKG2A, KIRs recognizing MHC-I) provide negative regulation by suppressing activating signals to maintain immune homeostasis.^88^^,^^89^

YTHDF2 can regulate the cytotoxic activity of NK cells and play an important role in tumor immunity (Table 1). In addition, it is a necessary regulatory factor for IL15-mediated NK cell survival.^51^ Mechanistically, YTHDF2 forms a STAT5-positive feedback loop by degrading TARDBP mRNA, enhancing NK cell survival and proliferation.^51^ Moreover, YTHDF2 deficiency impairs NK cell antitumor and antiviral activity.^51^ In the colorectal cancer TME, reduced SMAD4 expression in NK cells suppresses downstream YTHDF2 and the activating receptor NKG2D.^52^ When SMAD4 is overexpressed, the expression of downstream YTHDF2 can be up-regulated and the expression of the activating receptor NKG2D on the surface of NK cells can be promoted, thus significantly boosting NK cell cytotoxicity.^52^ Similarly, YTHDF2 overexpression in hepatocellular carcinoma (HCC) enhances NK cell cytotoxicity, inhibiting HCC proliferation, migration, and invasion.^53^ Conversely, elevated LINC00707 in HCC tissues promotes YTHDF2 ubiquitination and degradation, reducing NK cell cytotoxicity and accelerating HCC progression.^53^

In conclusion, YTHDF2 overexpression represents a promising therapeutic target for enhancing NK cell cytotoxicity and promoting antitumor immunity. Current evidence is limited to colorectal and hepatocellular carcinomas, with notable research gaps regarding other cancers, antiviral/antibacterial immunity, and graft-versus-host disease. Future studies should employ single-cell multi-omics and spatial transcriptomics techniques to elucidate NK cell dysfunction mechanisms, thereby informing therapeutic development.

## The role of YTHDF2 in T-cell immunity

T cells also play a crucial role in inflammatory responses, especially in the tumor microenvironment. Attracted by tumor-derived chemokines, they enhance antitumor immunity through cytotoxic particle and cytokine secretion.^90^^,^^91^ However, in some inflammatory diseases, excessive recruitment of T cells can drive the excessive secretion of inflammatory mediators, which instead promotes the progression of inflammation and aggravates tissue damage.^92^^,^^93^ Functionally classified subgroups include cytotoxic (CD8^+^ T cells), helper (CD4^+^ T cells: Th1/Th2/Th17), regulatory (Treg), and specialized populations (γδ T/Trm/NKT cells), which collaborate with inflammatory monocytes to coordinate immune responses.

YTHDF2 promotes antitumor immunity by regulating tumor chemokine secretion to drive CD8^+^ T-cell recruitment and infiltration (Table 1). In liver cancer, YTHDF2 stabilizes CX3CL1 expression to mediate CD8^+^ T-cell recruitment.^45^ Similarly, in colorectal cancer, KRT17 triggers YTHDF2 degradation via the ubiquitin–proteasome system, thus reducing CXCL10 mRNA decay. Elevated CXCL10 secretion then recruits CD8^+^ T cells into tumors, inhibiting tumor progression.^46^ In addition, YTHDF2 further modulates tumor surface receptors (PD-L1, RIG-I) to increase CD8^+^ T-cell infiltration. In bladder cancer (BLCA), YTHDF2 deficiency prevents its binding to DDX58 mRNA (encoding RIG-I), increasing RIG-I expression on tumor cells and promoting CD8^+^ T-cell-mediated tumor suppression.^44^ Conversely, ALKBH5 deficiency enriches m^6^A sites at PD-L1 mRNA’s 3′UTR; YTHDF2 recognition induces PD-L1 degradation, depleting this receptor on intrahepatic cholangiocarcinoma cells and enhancing T-cell infiltration and cytotoxicity.^64^

Of note, YTHDF2 can directly affect the proliferation, survival, and cytotoxicity of T cells (Table 1). In CD8^+^ T cells, YTHDF2 deficiency induces mitochondrial dysfunction and elevates IKZF1/3 transcription, leading to chromatin inactivation, reduced cell survival, and impaired antitumor immunity.^71^ Among regulatory T cells (Tregs), which mediate immunosuppression and tumor evasion, YTHDF2 knockout increases the expression of the NF-κB negative regulator TNFAIP3, promoting Treg apoptosis that attenuates immunosuppressive function and reduces tumor growth.^47^ Furthermore, YTHDF2 drives CD4^+^ T-cell recruitment to inflammatory sites, exacerbating disease progression.^67^ YTHDF2 cooperates with WTAP to degrade CES2 mRNA, inhibiting intestinal epithelial differentiation and mediating CD4^+^ T-cell infiltration in ulcerative colitis, thereby accelerating pathogenesis.^67^

In summary, YTHDF2 modulates tumor immunity through dual mechanisms: regulating T-cell recruitment via tumor chemokine secretion and surface receptor expression, while directly controlling T-cell proliferation, survival, and cytotoxicity.^46^^,^^67^^,^^90^^,^^91^ However, YTHDF2-mediated T-cell recruitment constitutes a double-edged sword. While enhancing tumor immunity, it also intensifies the inflammatory response in inflammatory disease tissues.^90^, ^91^, ^92^, ^93^ Consequently, developing YTHDF2-targeted therapeutics requires rigorous validation across disease contexts while preventing excessive immune responses that cause tissue damage and metabolic dysregulation.

## The role of YTHDF2 in B-cell immunity

B cells play a central role in both inflammatory and immune responses. Under inflammatory conditions, innate immune cells activate naïve B cells through the release of cytokines such as IL4 and IL6.^94^, ^95^, ^96^ These activated B cells then migrate to the germinal centers of secondary lymphoid organs, where they further differentiate into plasma cells and memory B cells.^97^ Plasma cells combat pathogens by secreting large quantities of high-affinity immunoglobulins, which form immune complexes and amplify the inflammatory process. In contrast, regulatory B cells help suppress overactive immune responses by producing anti-inflammatory factors like IL-10 and TGF-β, thereby mitigating tissue damage caused by excessive inflammation.^98^ Thus, B cells exert a dual regulatory influence in inflammatory settings and serve as key players in maintaining immune homeostasis. Dysregulation of B-cell function is closely associated with the development of chronic inflammatory diseases.

YTHDF2 modulates B-cell function by recognizing m^6^A or m^5^C sites on target genes, thereby promoting cancer progression and enhancing tumor drug resistance.^40^^,^^42^^,^^72^ In B-cell malignancies, YTHDF2 is highly overexpressed and facilitates tumor survival and immune evasion through two primary mechanisms. First, YTHDF2 recognizes m^5^C-modified mRNAs encoding F-type ATP synthase subunits and recruits poly(A)-binding protein C1 (PABPC1) to stabilize these transcripts. This significantly boosts ATP synthesis efficiency and provides the energy required for rapid cancer cell proliferation. Second, YTHDF2 binds to m^6^A-modified CD19 and MHC class II mRNAs, promoting their degradation. This reduces the visibility of tumor cells to the immune system and helps them to evade immunotherapeutic targeting.^42^ Furthermore, YTHDF2 is markedly up-regulated in diffuse large B-cell lymphoma (DLBCL). It binds to the m^6^A site within the 3′ UTR of ACER2 mRNA, stabilizing it and leading to increased ACER2 expression. This elevation triggers ceramide catabolism, subsequently activating the PI3K/AKT and ERK signaling pathways. The resulting up-regulation of Bcl2 inhibits apoptosis in DLBCL cells and accelerates disease progression.^40^ Additionally, highly expressed YTHDF2 recognizes the m^6^A site on C1qA mRNA and down-regulates its expression, contributing to the development of rituximab resistance in DLBCL.^72^

Further, YTHDF2 plays a key regulatory role in the early development of B cells.^99^, ^100^, ^101^ It is essential for the differentiation of progenitor B cells into large pre-B cells through mediating the decay of m^6^A-modified mRNAs. Loss of YTHDF2 function results in a significant reduction in splenic B-cell numbers.^100^ Furthermore, YTHDF2 critically influences germinal center B cells (glucocorticoid B cells) by suppressing the expression of genes associated with plasma cell formation^99^ and those involved in mitochondrial oxidative phosphorylation,^101^ thereby promoting the differentiation of glucocorticoid B cells.

In summary, YTHDF2 is a crucial regulator of early B-cell development. However, its aberrant overexpression contributes to the progression of B-cell malignancies and enhances drug resistance in DLBCL. Thus, YTHDF2 has distinct and seemingly contrasting effects on B-cell function. Current research on YTHDF2 in B cells has primarily focused on malignant transformation and early developmental stages. Further investigations are needed to elucidate the functional diversity and underlying mechanisms of YTHDF2 across different B-cell subsets, such as regulatory B cells and plasma cells.

## Molecular mechanisms of YTHDF2 in inflammation

YTHDF2 critically regulates inflammation through immune cell modulation. In this section, we discuss its mechanistic roles in inflammatory responses. YTHDF2 governs core inflammatory processes, including NLRP3 inflammasome activation and key signaling pathways (NF-κB, MAPK, and JAK–STAT) by modulating RNA stability. This regulation triggers cellular inflammation and apoptosis, which significantly impact various physiological and pathological processes.

## YTHDF2 regulation of the NF-κB signaling pathway

NF-κB, a ubiquitous eukaryotic transcription factor, is activateds via TLRs or cytokine receptors (e.g., TNFαR) upon PAMP/DAMP recognition. This activation drives proinflammatory factor expression, chemokine production, and the synthesis of key enzyme (including iNOS and COX-2), mediating acute inflammation. However, persistent NF-κB signaling promotes chronic inflammation that accelerates cellular senescence, tissue damage, and cancer proliferation.^102^, ^103^, ^104^

**Figure 3:**
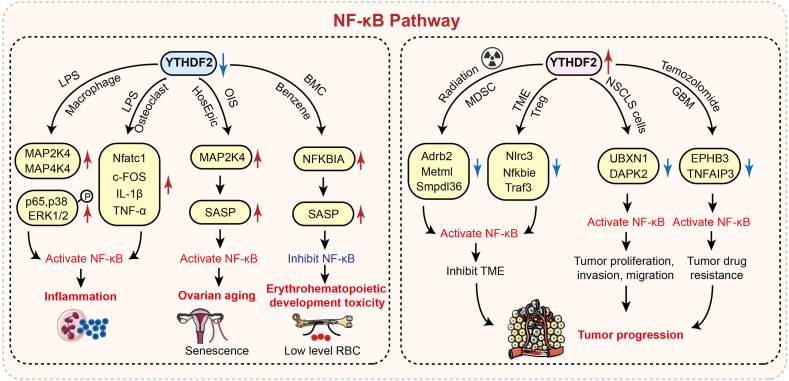
YTHDF2 modulates the NF-κB pathway. YTHDF2 modulates the NF-κB pathway by regulating downstream RNA targets, exacerbating inflammation, aging, hematopoietic toxicity, and tumor progression.

YTHDF2 deficiency activates NF-κB signaling by stabilizing MAP2K4 and MAP4K4 mRNAs, amplifying inflammatory responses (Fig. 3). In LPS-induced macrophages, YTHDF2 knockout elevates MAP2K4/MAP4K4 mRNA and enhances p65, p38, and ERK1/2 phosphorylation, activating both the MAPK and NF-κB pathways to exacerbate inflammation.^78^ Similarly, reduced YTHDF2 in diabetic retinal endothelial cells stabilizes MAP4K4 mRNA, triggering NF-κB activation that promotes endothelial dysfunction, microvascular abnormalities, and accelerated diabetic retinopathy in *db/db* mice.^105^ Furthermore, YTHDF2 knockout in LPS-stimulated osteoclasts increases osteoclastogenic transcription factors (Nfatc1, c-Fos) and proinflammatory cytokines (IL1β, TNFα), driving osteoclastogenesis and inflammatory bone disease progression.^106^

Notably, YTHDF2 can intervene in the tumor immune microenvironment by degrading mRNAs encoding negative regulators of NF-κB, thereby activating NF-κB signaling. Increased YTHDF2 expression in irradiation-induced myeloid-derived suppressor cells (MDSCs) degrades NF-κB regulatory mRNAs (Adrb2, Metml, Smpdl3b) and activates NF-κB to drive MDSC migration and suppress antitumor immunity.^29^ Furthermore, TNF-mediated YTHDF2 up-regulation in tumor-infiltrating Treg cells degrades negative regulators (Nlrc3, Nfkbie, and Traf3), thus activating NF-κB to inhibit Treg apoptosis and promote tumor progression through immunosuppression.^47^

In addition to modulating the tumor immune microenvironment, YTHDF2 also directly governs tumor cell proliferation, invasion, and migration. Studies have demonstrated that YTHDF2 cooperates with METTL3 to degrade the mRNAs of the NF-κB inhibitor UBXN1^107^ and the tumor suppressor DAPK2.^48^ This activates the NF-κB pathway, thereby amplifying tumor cell malignancy and accelerating progression. Moreover, YTHDF2 can promote drug resistance: in temozolomide-treated glioblastoma, elevated YTHDF2 binds and degrades EPHB3 and TNFAIP3 mRNAs via their 3′UTRs. This leads to reduced EPHB3/TNFAIP3 expression, which subsequently activates PI3K/AKT and NF-κB signaling and promotes chemotherapy resistance.^108^

Additionally, YTHDF2 promotes cellular senescence by regulating the NF-κB pathway and modulating the senescence-associated secretory phenotype (SASP). Reduced YTHDF2 expression elevates MAP2K4 mRNA levels, activating the NF-κB and MAPK pathways to increase SASP production and accelerate oncogene-induced senescence (OIS) ovarian aging.^109^ Furthermore, in benzene-exposed mouse bone marrow cells (BMCs), decreased YTHDF2 and METTL3 levels elevate the mRNA expression of NFKBIA, an NF-κB inhibitor, thereby suppressing NF-κB signaling and reducing SASP. This impairment delays macrophage recognition and clearance of senescent BMCs, ultimately contributing to benzene-induced hematopoietic defects.^110^

In summary, the NF-κB signaling pathway serves as a pivotal link between chronic inflammation and tumor development. YTHDF2, a key regulator of NF-κB signaling, participates in chronic inflammatory responses, exacerbates cellular senescence, and promotes tumor progression. Notably, the expression and downstream targets of YTHDF2 vary across microenvironments. Therefore, future studies should employ multi-omics approaches to delineate tissue- and cell-specific mechanisms of YTHDF2-mediated NF-κB regulation to provide a stronger foundation for advancing therapeutic strategies for inflammatory diseases and cancers.

## YTHDF2 regulation of the MAPK signaling pathway

The MAPK signaling pathway mediates cellular responses to external stimuli (including stress, pathogens, and cytokines) through its ERK, JNK, and p38-MAPK sub-pathways. It serves as a central regulator of inflammatory responses, proliferation, apoptosis, and cell survival.

**Figure 4:**
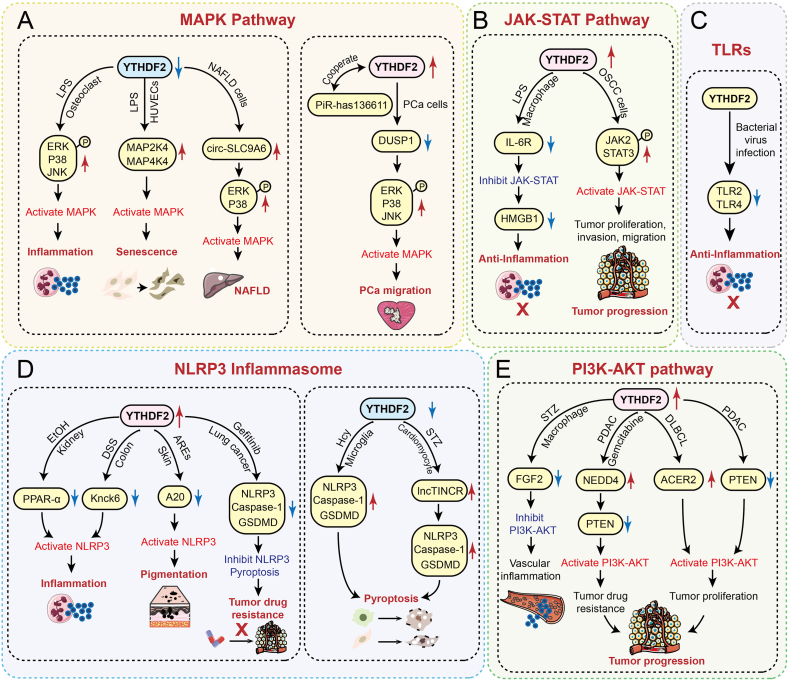
YTHDF2 modulates MAPK, JAK–STAT, PI3K-AKT, TLRs, and the NLRP3 inflammasome. YTHDF2 modulates inflammation, aging, and tumors by regulating the **(A)** MAPK, **(B)** JAK–STAT, and **(C)** PI3K-AKT pathways; **(D)** TLRs; and **(E)** the NLRP3 inflammasome.

Low expression of YTHDF2 promotes inflammatory responses by activating the MAPK signaling pathway (Fig. 4A). YTHDF2 silencing promotes the phosphorylation of ERK, p38, and JNK in LPS-induced osteoclasts, activating MAPK signaling and exacerbating osteoclast inflammation.^106^ Parallel observations in LPS-stimulated macrophages have shown that YTHDF2 knockdown similarly enhances p38 and ERK1/2 phosphorylation, amplifying inflammatory responses.^78^

Low YTHDF2 expression promotes cellular senescence by activating the MAPK signaling pathway and elevating SASP levels. In LPS-stimulated human umbilical vein endothelial cells, decreased YTHDF2 levels lead to MAP2K4 and MAP4K4 mRNA stabilization and activate the MAPK pathway to increase SASP production and accelerate senescence.^111^ Similarly, in oncogene-induced senescent human ovarian surface epithelial cells, YTHDF2 downregulation stabilizes MAP2K4 mRNA, triggering MAPK-mediated SASP expression and exacerbating cellular aging.^109^

Furthermore, reduced YTHDF2 expression disrupts lipid homeostasis by activating the MAPK signaling pathway. Decreased YTHDF2 in non-alcoholic fatty liver disease (NAFLD) tissues impairs circ-SLC9A6 degradation. This circRNA encodes an abundant SLC9A6-126aa protein, which translocates to the nucleus and binds the CD36 promoter. Subsequent CD36 transcription elevates p38/ERK phosphorylation, activating MAPK signaling to drive lipid metabolic dysfunction and accelerate NAFLD progression.^112^

However, in prostate cancer (PCa) tissues, when YTHDF2 is highly expressed, it interacts with piR-has-136611 to degrade the mRNA of the MAPK inhibitor DUSP1. This further elevates ERK, p38, and JNK phosphorylation, which activates the MAPK pathway to drive PCa cell metastasis and accelerate disease progression.^113^

Overall, reduced YTHDF2 expression leads to MAPK pathway activation, which promotes inflammatory responses, exacerbates cellular senescence, and disrupts lipid homeostasis. Conversely, elevated YTHDF2 expression can promote cancer progression through MAPK pathway activation.

## YTHDF2 regulation of the JAK–STAT signaling pathway

The JAK–STAT pathway comprises Janus kinases (JAKs) and signal transducers and activators of transcription (STATs). When cytokines or growth factors bind to membrane receptors, the JAK family proteins are activated, which then mediate the phosphorylation of tyrosine in the binding receptor. This promotes the dissociation of STAT from the receptor to form dimers and its translocation to the nucleus, thereby initiating the transcription of inflammatory factors and chemokines. JAK–STAT dysregulation is implicated in chronic inflammatory diseases, including rheumatoid arthritis, psoriasis, inflammatory bowel disease, and tumors.^114^ YTHDF2 can precisely regulate this pathway.

YTHDF2 overexpression suppressed the JAK–STAT pathway in LPS-stimulated macrophages, attenuating inflammation (Fig. 4B). Mechanistically, YTHDF2 recognizes and binds to m^6^A sites on the IL6R mRNA to promote its degradation, thus inhibiting JAK2/STAT1 activation. This ultimately reduces the extracellular release of the proinflammatory factor HMGB1, exerting anti-inflammatory effects that mitigate disease severity in septic mice.^115^

Furthermore, the overexpression of YTHDF2 can exacerbate cancer progression through the activation of the JAK–STAT signaling pathway. In oral squamous cell carcinoma (OSCC) tissues, YTHDF2 expression was significantly up-regulated, leading to increased phosphorylation levels of JAK2 and STAT3 in OSCC cells. This subsequently resulted in the activation of the JAK–STAT pathway, which facilitates the proliferation, metastasis, and invasion of OSCC cells, thereby accelerating the progression of OSCC.^116^

In conclusion, as a key regulator of the JAK–STAT signaling pathway, YTHDF2 exhibits cell type-specific functions. In immune cells, it exerts anti-inflammatory effects by negatively regulating the JAK–STAT pathway through the degradation of receptor mRNAs such as IL6R.^115^ In cancer cells, however, high expression of YTHDF2 promotes activation of the JAK–STAT pathway, thereby driving malignant progression.^116^ This dual regulatory role underscores the complexity of YTHDF2 in disease contexts and supports its potential as a target for precise therapeutic intervention.

## YTHDF2 regulates the NLRP3 inflammasome

The nod-like receptor protein 3 (NLRP3) inflammasome is a cytoplasmic multiprotein complex comprising the intracellular pattern recognition receptor NLRP3, the adaptor apoptosis-associated speck-like protein (ASC), and the effector protease caspase-1.^117^ As a key component of the innate immune system, the NLRP3 inflammasome is present in immune cells such as macrophages and dendritic cells as well as some non-immune cells. This inflammasome assembles and is activated in response to the sensing of PAMPs and DAMPs. Activated caspase-1 cleaves downstream proinflammatory cytokine precursors, such as pro-IL1β and pro-IL18, converting them into their biologically active mature forms (IL1β and IL18), thereby driving inflammation. Caspase-1 also cleaves the pore-forming protein gasdermin D (GSDMD), mediating pyroptosis through cell membrane pore formation.^118^

YTHDF2 collaborates with other m^6^A regulatory factors to activate the NLRP3 inflammasome, amplifying inflammatory responses (Fig. 4D). In mouse models of alcoholic kidney injury, YTHDF2 expression increases, while FTO expression decreases.^119^ YTHDF2 acts in conjunction with FTO to reduce the mRNA stability of the NLRP3 negative regulator PPAR-α,^120^ thereby activating the NLRP3 inflammasome, elevating inflammatory factors in renal tubular epithelial cells, and exacerbating kidney injury.^119^ Additionally, YTHDF2 cooperates with METTL3 to promote NLRP3 inflammasome activation. Mechanistically, this collaboration enhances KCNK6 mRNA stability and activates KCNK6 transcription through histone acetylation. KCNK6 potentiates potassium channel activity, driving NLRP3 inflammasome activation and aggravating colitis and colon cancer progression.^74^ Moreover, YTHDF2 recognizes m^6^A sites on the anti-inflammatory factor A20 and suppresses its expression, consequently activating the NLRP3 inflammasome, NF-κB, and MAPK inflammatory pathways and promoting advanced glycation end product-induced skin melanin deposition.^121^

Furthermore, YTHDF2 plays a critical role in NLRP3 inflammasome activation-induced pyroptosis. In homocysteine (Hcy)-treated microglia, reduced YTHDF2 expression enhances NLRP3 mRNA stability, thus activating the NLRP3/caspase-1/GSDMD pathway and exacerbating microglial pyroptosis.^80^ Similarly, decreased METTL14 expression in diabetic cardiomyocytes diminishes YTHDF2 binding to lncRNA TINCR. This slows lncRNA TINCR decay, leading to up-regulated NLRP3 mRNA expression and promoting cardiomyocyte pyroptosis.^122^ Conversely, in gefitinib-resistant lung cancer cells, elevated levels of YTHDF2, METTL3, and LINC00969 cooperate to degrade NLRP3 mRNA, preventing caspase pyroptosis pathway activation and contributing to drug resistance.^50^

In conclusion, the NLRP3 inflammasome serves as a critical hub linking cellular inflammatory responses to pyroptosis. YTHDF2 finely modulates the activation of the NLRP3 inflammasome via m^6^A epigenetic modification. In the future, it may serve as a pivotal therapeutic target for inflammatory diseases by targeting the NLRP3 inflammasome.

## YTHDF2 regulation of the TLR signaling pathway

TLRs, a class of pattern recognition receptors, are widely distributed on immune and epithelial cells. Similar to NLRP3 inflammasome activation, TLRs recognize pathogen-associated molecular patterns (PAMPs) and DAMPs, triggering downstream signaling that promotes cytokine and chemokine production to activate anti-infection immunity and inflammatory responses.^123^ Upon activation, TLR signaling primarily occurs through two classic pathways: the MyD88-dependent or TRIF-dependent routes. Most TLRs (e.g., TLR2, TLR4, and TLR5) utilize the adaptor protein MyD88 to recruit interleukin-1 receptor-associated kinase (IRAK), activate TRAF6, and ultimately induce NF-κB and AP-1 transcription factors. This drives acute inflammation by stimulating proinflammatory factors (e.g., TNF-α, IL-6, and IL-1β) and chemokines (e.g., CXCL8).^124^^,^^125^ Additionally, specific TLRs (notably TLR3 and TLR4) signal through TRIF to activate interferon regulatory factor 3 (IRF3) and NF-κB, promoting type I interferons (IFN-α/β) and inflammatory mediators, which is critical for antiviral immunity.^126^

YTHDF2 modulates inflammatory responses following bacterial and viral infections by regulating TLR mRNA stability (Fig. 4C). Under normal conditions, DDX5 forms complexes with METTL3 and METTL14 to enhance TLR2/4 mRNA methylation.^127^ YTHDF2 recognizes these methylation sites and degrades the mRNA, thereby suppressing inflammation. However, during bacterial infection, Hrd1 recruits DDX5 for ubiquitin–proteasomal degradation, disrupting complex formation with METTL3/METTL14. This reduces TLR2/4 mRNA methylation, preventing YTHDF2 recognition and subsequent degradation. Consequently, accumulated TLR2/4 mRNA activates TLR signaling, triggering inflammation.^127^ Conversely, in swine fever virus-infected cells, elevated METTL14 expression increases TLR4 m^6^A methylation.^128^ YTHDF2 recognizes these sites and degrades TLR4 mRNA, thereby blocking NF-κB inflammatory signaling and inhibiting host immune responses to promote viral infection.^128^ As core hubs linking innate immunity and inflammation, precise TLR regulation is crucial for balancing host defense and immunopathology. YTHDF2 represents a novel mechanism for such TLR modulation.

## YTHDF2 regulation of the PI3K–AKT signaling pathway

The PI3K–AKT signaling pathway critically regulates inflammatory responses, cell proliferation, survival, migration, and apoptosis. Its abnormal activation is implicated in diverse pathologies including cancer, inflammatory disorders, and metabolic diseases.^129^, ^130^, ^131^ This pathway operates through phosphatidylinositol 3-kinase (PI3K), which catalyzes the generation of the second messenger PIP3. PIP3 subsequently activates protein kinase B (AKT), initiating downstream signaling cascades.

YTHDF2 promotes inflammatory responses by activating the PI3K/AKT pathway (Fig. 4E). In a Sprague Dawley rat model of ischemic stroke (tMCAO), YTHDF2 expression was significantly increased. It elevates brain inflammation by negatively regulating Matrilin-3 and activating PI3K/AKT signaling while also exacerbating ischemic stroke progression by reducing blood–brain barrier protein expression.^132^ While PI3K/AKT pathway activation is typically associated with proinflammatory responses in most cells, Yan et al revealed a contrasting role. They found that in diabetic retinopathy macrophages, increased YTHDF2 combined with reduced FTO levels promotes FGF2 mRNA degradation, thereby suppressing PI3K/AKT pathway activation.^66^ This inhibition drives macrophage polarization toward the M1 phenotype, worsening diabetic retinopathy inflammation and microvascular dysfunction.^66^ Thus, PI3K/AKT pathway activation exhibits bidirectional regulation of inflammation, likely depending on the disease stage and the cellular microenvironment. For example, acute disease phases may utilize this pathway to promote pathogen-clearing inflammation, whereas its activation in chronic inflammatory conditions could instead suppress inflammation to prevent tissue damage.

Furthermore, YTHDF2 promotes tumor progression by activating the PI3K–AKT signaling pathway. High YTHDF2 expression induces tumor cell drug resistance through this pathway.^108^^,^^133^ Mechanistically, YTHDF2 binds to the 3′UTRs of EPHB3 and TNFAIP3 mRNAs, thus promoting their degradation and subsequent PI3K–AKT activation, which induces temozolomide resistance in glioblastoma.^108^ Similarly, in pancreatic cancer, YTHDF2 enhances NEDD4 mRNA stability and reduces PTEN expression, activating PI3K–AKT to confer gemcitabine resistance.^133^ Moreover, YTHDF2 drives tumor proliferation, invasion, and metastasis via the PI3K–AKT pathway. In DLBCL, elevated YTHDF2 stabilizes ACER2 mRNA by binding to its m^6^A-modified 3′UTR. Increased ACER2 triggers ceramide catabolism, activating PI3K/AKT and ERK pathways, up-regulating BCL2, suppressing apoptosis, and promoting tumor proliferation.^40^ Likewise, in pancreatic ductal adenocarcinoma, hsa_circ_0007590 binds PTBP1 to up-regulate YTHDF2, which degrades PTEN mRNA, activating the PI3K–Akt pathway.^134^ This induces the Warburg effect, fueling cancer cell proliferation, invasion, and metastasis.^134^ Additionally, YTHDF2 cooperates with METTL3 to activate PI3K/AKT signaling and promote cancer cell proliferation.^135^^,^^136^

In summary, the PI3K–AKT pathway acts as the central processing unit of the cellular signaling network, where its precise regulation is vital for maintaining life activities. YTHDF2, a key regulator of this pathway, contributes to inflammatory disease and tumor progression, offering novel insights for PI3K–AKT-targeted drug development.

## YTHDF2 in inflammatory diseases

YTHDF2 contributes to inflammatory responses in multiple diseases by modulating downstream RNA stability, thereby regulating inflammation-related immune cell functions and the activation of inflammatory pathways and inflammasomes. Under inflammatory conditions, aberrant YTHDF2 expression in non-immune cells, including epithelial cells, endothelial cells, and cardiomyocytes, further exacerbates the inflammatory process. This section details the roles of YTHDF2 across various inflammatory diseases (Table 2).

**Table 2:** The functions of YTHDF2 in inflammatory diseases.

Disease	Model	Cooperative m^6^A regulators	Target	Biological outcome	Ref
COVID-19	SARS-CoV-2 infected cells	/	Viral RNA ↓	Promote the decay of viral RNA thereby suppressing COVID-19	137
IPF	Bleomycin induced IPF mice/A549	METTL3	TSC1 ↓	Activating AKT/mTOR thereby promoting IPF	138
IBD	Dextran sulfate sodium induced IBD mice/NCM460/Caco-2	METTL3, ALKBH5	KLF4 ↓	Disrupting intestinal homeostasis thereby promoting IBD	139
UC	Dextran sulfate sodium induced UC mice/primary enterocytes/BMDM	WTAP	CES2 ↓	Aggravating inflammation thereby promoting UC	67
AIH	YTHDF2 MDSC-deficient mice/ConA induced hepatitis mice	/	RXRα ↑	YTHDF2 MDSC-deficient mice can alleviate ConA-induced hepatitis	140
Psoriasis	Imiquimod induced psoriasis mice/HaCaT	/	DKK3 ↓	Activating Wnt pathway thereby promoting psoriasis	141
OA	Medial meniscal instability surgery on mice/primary mouse chondrocytes	/	SREBF2 ↑	Ubiquitination degradation of YTHDF2 up-regulating SREBF2 thereby promoting OA	142
Periodontitis	Ectopic bone formation assay conducted on mice/OCCM-30	FTO	Runx2 ↓	FTO deficiency induced Runx2 decay by YTHDF2 thereby promoting periodontitis	143
DR	Streptozotocin induced diabetic mice/RMEC/rMC	/	ITGB1 ↑	Down-regulated YTHDF2 inhibits the decay of ITGB1 thereby activating FAK/PI3K/AKT thus promoting DR	144
	db/db diabetic mice/HRMECs	/	MAP4K4 ↑	Down-regulated YTHDF2 inhibits the decay of MAP4K4 thereby activating NF-κB thus promoting DR	105
Alcoholic kidney injury	Alcohol-induced kidney injury mice/HK2	FTO	PPAR-α ↓	YTHDF2 induced PPAR-α decay cooperating with low-expressed FTO thereby activating NLRP3 thus promoting alcoholic kidney injury	119
Sepsis	LPS induced sepsis mice/PBMC/RAW264.7	/	IL6R ↓	Inhibiting JAK2/STAT1 thereby reducing HMGB1 thus promoting sepsis	115
SIC	LPS induced SIC mice/primary mouse cardiomyocyte	RBM15	SOX18 ↓	YTHDF2 cooperating with RBM15 to reduce SOX18 thereby promoting pyroptosis of myocardial cells	145
	H9C2	METTL3	SLC7A11 ↓	YTHDF2 cooperating with METTL3 to reduce SLC7A11 leading to ferroptosis of myocardial cells	146
	FTO deficient mice	FTO	BNIP3 ↓	FTO cooperating with YTHDF2 to reduce BNIP3 inhibiting mitochondrial autophagy thus promoting myocardial cells apoptosis	147
Bacterial infection	THP-1	/	KDM6B ↑	Deficiency of YTHDF2 up-regulate KDM6B to enhance inflammation	148

## YTHDF2 in respiratory system diseases

### COVID-19

Inflammatory responses play central roles in the development of respiratory diseases and serve as key drivers of chronic disease progression and complications. SARS-CoV-2 infection triggers a cytokine storm (characterized by surges in IL6, IFNγ, and GM-CSF levels) that damages the pulmonary vasculature and disrupts the alveolar–capillary barrier. This leads to diffuse pulmonary fibrin exudation, refractory hypoxemia, and severe acute respiratory syndrome. YTHDF2 serves an inhibitory function in COVID-19 by recognizing m^6^A modification sites on SARS-CoV-2 RNA and promoting viral RNA decay.^137^ Specifically, reduced intracellular H3K27-me3 levels increase m^6^A modifications on viral RNA, recruiting YTHDF2 to bind to viral transcripts. This binding destabilizes viral RNA, thereby suppressing COVID-19 pathogenesis.^137^

### Idiopathic pulmonary fibrosis

Repeated micro-injuries activate alveolar epithelial cells to secrete profibrotic factors like TGF-β and PDGF, driving fibroblast-to-myofibroblast transformation, excessive extracellular matrix deposition, and inflammatory mediator release, which are hallmark features of idiopathic pulmonary fibrosis. TSC1 critically regulates cell growth, proliferation, and development. YTHDF2 cooperates with METTL3 to degrade TSC1 mRNA in lung epithelial cells, thereby activating the AKT/mTOR pathway, facilitating epithelial–mesenchymal transition, and ultimately accelerating idiopathic pulmonary fibrosis progression.^138^

Taken together, YTHDF2 plays a dual role in respiratory diseases. In COVID-19, it exerts a protective antiviral effect by recognizing and degrading m6A-modified viral RNA.^137^ In idiopathic pulmonary fibrosis; however, YTHDF2 promotes the degradation of TSC1 mRNA and activates the AKT/mTOR pathway, thereby exacerbating the fibrotic process.^138^ This contrast further highlights that the function of YTHDF2 is highly context-dependent.

## YTHDF2 in digestive system diseases

### Inflammatory bowel disease

Inflammatory bowel disease (IBD), encompassing ulcerative colitis and Crohn’s disease, is characterized by intestinal epithelial barrier dysfunction and chronic inflammation. Intestinal epithelial cells and AJC proteins critically maintain barrier homeostasis. YTHDF2 collaborates with METTL3 and ALKBH5 to destabilize KLF4 mRNA. As a transcription factor regulating AJC expression, reduced KLF4 levels decrease AJC proteins, elevate ROS and proinflammatory factors in epithelial cells, increase epithelial apoptosis, and accelerate IBD progression.^139^ Additionally, abnormal epithelial differentiation and extensive immune cell infiltration further exacerbate IBD. YTHDF2 cooperates with WTAP to degrade CSE2 mRNA, inhibiting intestinal epithelial differentiation in mice, polarizing macrophages toward the M1 phenotype, and recruiting CD4^+^ T cells into ulcerative colitis tissues, which collectively accelerate IBD pathogenesis.^67^

### Hepatitis

Hepatitis is characterized by hepatocyte damage, necrosis, and extensive inflammatory cell infiltration. Untreated, it can progress to liver fibrosis, cirrhosis, or hepatocellular carcinoma. Elevated YTHDF2 expression promotes hepatitis progression.^77^^,^^140^ YTHDF2 degrades RXRα mRNA, impairing the immunosuppressive function of MDSCs.^140^ This triggers T-cell proliferation and exacerbates concanavalin A (ConA)-induced hepatitis. MDSC-specific YTHDF2 knockout enhances immunosuppressive function and alleviates ConA-induced liver injury.^140^ Glucocorticoids exert potent anti-inflammatory effects; however, in LPS-stimulated Kupffer cells, up-regulated YTHDF2 reduces glucocorticoid receptor mRNA stability. Consequently, glucocorticoid receptors cannot bind glucocorticoids, inhibiting the transcription of anti-inflammatory factors such as glucocorticoid-induced leucine zipper (GILZ), ultimately amplifying hepatic inflammation.^77^

By targeting distinct cell types and key molecules, YTHDF2 contributes to the exacerbation of inflammatory processes in both the gastrointestinal tract and the liver. In IBD, it promotes pathogenesis by impairing intestinal epithelial barrier integrity, as it targets molecules such as KLF4 and CSE2, and disrupts the local immune microenvironment by promoting M1 macrophage polarization and T-cell recruitment.^67^^,^^139^ In hepatitis, YTHDF2 amplifies inflammatory liver injury through a dual mechanism: it weakens the immunosuppressive function of MDSCs and antagonizes the anti-inflammatory signaling of glucocorticoid receptors.^77^^,^^140^ These findings underscore YTHDF2 as a central player linking epithelial dysfunction and immune dysregulation, highlighting its context-dependent roles across different inflammatory diseases.

## YTHDF2 in inflammatory diseases of the skin, joints, and bones

### Psoriasis

Psoriasis is a chronic inflammatory skin disease caused by immune imbalance and excessive activation of inflammatory factors, usually leading to abnormal proliferation and differentiation disorders of epidermal keratinocytes and skin inflammatory responses. YTHDF2 is abnormally expressed in the skin lesion tissues of patients with psoriasis.^141^ Furthermore, in the *in vitro* cell model of psoriasis constructed by HaCaT cells induced by the M5 combination (human recombinant proteins IL1α, IL17A, IL22, TNFα, and oncostatin M), and in the *in vivo* mouse model of psoriasis treated with imiquimod, the expression of YTHDF2 was abnormally increased.^141^ Mechanistically, YTHDF2 promotes the activation of the Wnt signaling pathway by degrading the mRNA of the Wnt signaling pathway inhibitor DKK3, thereby facilitating inflammatory responses and abnormal proliferation of epidermal cells and thus accelerating the progression of psoriasis.^141^

### Osteoarthritis and inflammatory bone diseases

Osteoarthritis (OA), a chronic degenerative inflammatory disease, significantly impacts elderly populations. Its pathogenesis involves multifaceted interactions, including mechanical injury, metabolic abnormalities, inflammatory responses, genetic factors, and imbalanced cell death patterns. YTHDF2 exacerbates OA progression by promoting chondrocyte ferroptosis and autophagy dysfunction.^142^^,^^149^ In OA models, the up-regulated ubiquitin ligase TRIM8 in chondrocytes enhances YTHDF2 ubiquitination. Reduced YTHDF2 expression impairs SREBF2 degradation, and subsequent SREBF2 upregulation promotes chondrocyte ferroptosis.^142^ Furthermore, YTHDF2 increases SETD7 expression in OA chondrocytes by stabilizing SETD7 mRNA, causing autophagy impairment that amplifies chondrocyte damage under inflammatory conditions.^149^

Notably, YTHDF2 exacerbates inflammatory bone diseases, including osteoporosis, rheumatoid arthritis, and periodontitis. Excessive osteoclast activity disrupts bone remodeling homeostasis, driving disease progression. YTHDF2 knockout in osteoclast precursors (RAW264.7 cells and bone marrow-derived macrophages) stabilizes mRNAs encoding osteoclastogenic transcription factors (Nfatc1, c-Fos) and elevates the proinflammatory cytokines IL1β and TNFα. This dual effect amplifies osteoclast formation and inflammatory responses, accelerating bone disease.^106^ Furthermore, YTHDF2 degrades Runx2 mRNA, a key cementum formation factor, impairing cementoblast differentiation and compromising periodontal tissue integrity.^143^

Overall, YTHDF2 plays key roles in various inflammatory diseases through tissue-specific and diverse regulatory mechanisms. In psoriasis, its elevated expression promotes inflammation and abnormal cell proliferation by activating the Wnt signaling pathway.^141^ In osteoarthritis, alterations in its expression—modulated by ubiquitination-mediated degradation or mRNA stability—exacerbate chondrocyte damage through the ferroptosis and autophagy pathways, respectively.^142^^,^^149^ In a broader spectrum of inflammatory bone disorders, YTHDF2 disrupts bone homeostasis by regulating key transcription factors involved in osteoclast formation and factors critical for cement–bone differentiation.^106^ Together, these findings establish YTHDF2 as a pivotal molecule linking post-transcriptional regulation to tissue-specific inflammatory pathology.

### Diabetic retinopathy

Diabetic retinopathy (DR), a serious microvascular complication of diabetes, features retinal vascular damage and can cause vision loss. YTHDF2 deficiency drives DR pathogenesis.^144^ In streptozotocin (STZ)-induced diabetic mice, downregulated KAT1 protein, which promotes histone acetylation in the YTHDF2 promoter to activate transcription, suppresses YTHDF2 expression.^144^ This impairs ITGB1 mRNA degradation, activating the FAK/PI3K/AKT pathway to stimulate retinal microvascular endothelial cell (RMEC) proliferation and neovascularization, accelerating DR progression.^144^ Additionally, reduced YTHDF2 stabilizes MAP4K4 mRNA, triggering NF-κB pathway activation that decreases the retinal tight junction protein Claudin-5, compromising vascular integrity and further advancing DR.^105^

### Alcohol-induced kidney injury

Alcohol-induced renal injury arises from multiple pathways, including metabolite (acetaldehyde) cytotoxicity, inflammatory responses, oxidative stress, and lipid metabolism disorders, which collectively cause renal impairment. In experimental models of alcoholic kidney injury, YTHDF2 cooperates with lowly expressed FTO to degrade PPAR-α mRNA, thereby activating the NLRP3 inflammasome and promoting inflammation in renal tubular epithelial cells, ultimately exacerbating kidney damage.^119^

## YTHDF2 in infectious diseases

### Sepsis

Sepsis, a systemic inflammatory response syndrome triggered by microbial infections, induces multiple organ dysfunction. YTHDF2 recognizes the m^6^A modification site on the IL6R mRNA and reduces its stability, thereby inhibiting JAK2/STAT1 signaling. This suppression curbs both HMGB1 release and sepsis-induced inflammation.^115^

Sepsis-induced inflammatory mediators cause myocardial damage, leading to sepsis-induced cardiomyopathy, which rapidly impairs cardiac function. In LPS-induced cardiomyocyte models, up-regulated YTHDF2 cooperates with RBM15 to destabilize SOX18 mRNA. Reduced SOX18 elevates PTX3 expression, triggering NLRP3 inflammasome formation and cardiomyocyte pyroptosis.^145^ YTHDF2 can collaborate with METTL3 to degrade SLC7A11 mRNA, induce ferroptosis in cardiomyocytes, and accelerate cardiomyopathy progression.^146^ Furthermore, YTHDF2 can inhibit mitochondrial autophagy in cardiomyocytes, increase ROS in cardiomyocytes, and induce cardiomyocyte apoptosis.^147^ Mechanistically, after FTO silencing, the m^6^A modification level of the mitochondrial autophagy-related gene BNIP3 increases. YTHDF2 recognizes the m^6^A-modified site of BNIP3 and promotes the degradation of its mRNA, thereby suppressing mitochondrial autophagy in cardiomyocytes and exacerbating apoptosis in sepsis-induced cardiomyopathy cardiomyocytes.^147^

### Bacterial infection

Bacterial infections trigger diverse diseases with clinical manifestations dependent on the inflammatory intensity and pathogen characteristics. Upon bacterial invasion, PAMPs are recognized by pattern recognition receptors on innate immune cells, activating the NF-κB and MAPK signaling pathways. This drives proinflammatory cytokine and chemokine release, collectively promoting vasodilation, increased endothelial permeability, and recruitment of neutrophils and macrophages to infection sites. These cells eliminate pathogens through phagocytosis, ROS release, and antimicrobial peptide secretion. YTHDF2 deficiency potentiates inflammatory responses during bacterial infection by up-regulating KDM6B expression.^148^ Elevated KDM6B demethylates histone H3K27me3 at promoters of proinflammatory genes (IL6, IL12B), enhancing their transcription and exacerbating inflammation.^148^

Overall, YTHDF2 plays a complex and context-dependent regulatory role in infectious diseases, with contrasting effects in different environments. In systemic sepsis, it exerts anti-inflammatory and protective effects by suppressing the IL6R/JAK–STAT pathway.^115^ However, in sepsis-induced cardiomyopathy, a complication of sepsis, high YTHDF2 expression exacerbates cardiac injury by inducing multiple forms of cardiomyocyte death, such as pyroptosis and ferroptosis, while also inhibiting autophagy.^145^, ^146^, ^147^ Furthermore, during bacterial infections, the absence of YTHDF2 amplifies the inflammatory response by enhancing the transcription of inflammatory factors through epigenetic mechanisms.^148^ These findings underscore that the role of YTHDF2 is highly dependent on the stage and tissue context of infection, positioning it as a key regulator in balancing host immune defense and inflammatory damage.

## Potential therapeutic agents targeting YTHDF2

**Figure 5:**
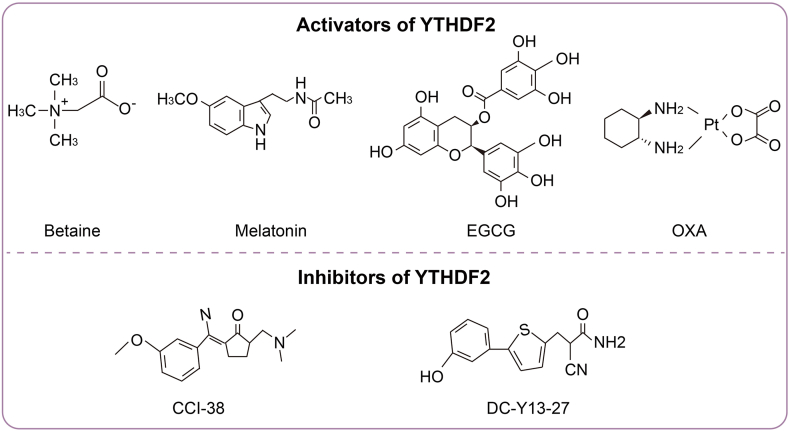
Chemical structures of YTHDF2 activators and inhibitors.

YTHDF2 critically regulates inflammatory diseases by modulating immune cell functions, inflammatory pathway activation, and inflammasome assembly. Targeting YTHDF2 offers novel therapeutic strategies for inflammatory conditions. This section summarizes existing YTHDF2 agonists and inhibitors (Fig. 5 and Table 3).

**Table 3:** Potential drugs targeting YTHDF2 for the treatment of inflammatory diseases.

Activators of YTHDF2						
Drug	FDA Approved	Category	Disease	Pre-clinical models	Relevant mechanisms	Ref
Betaine	Approved	Natural product	Dementia	SD rats injected with Hcy	Suppression of NLRP3/caspase-1/GSDMD-dependent microglial pyroptosis	80
Melatonin	NA	Natural product	Ovarian aging	OIS induced human ovarian surface epithelial cells	Suppressing the NF-κB pathway	109
EGCG	NA	Natural product	Obesity	IBMX, dexamethasone and insulin induced mouse 3T3-L1 precursor adipocytes	Decreasing CCNA2/CDK2 mRNA and protein stability, thereby inhibiting adipocyte differentiation	150
Oxaliplatin (OXA)	Approved	Synthetic compound	Liver cancer	*In vivo*: Orthotopic and metastatic liver tumor models	Enhancing Cx3cl1mRNA stability, thereby recruiting CD8^+^ T cells to increase tumor immunity	45
				*In vitro*: Hepatoma cell lines (Hepa1-6 and AML12)		
**Inhibitors of YTHDF2**						

CCI-38	NA	Synthetic compound	B Cell malignancies	*In vivo*: Ythdf2-specific knockout mice and immunodeficient mice	Reducing ATP synthesis in cancer cells and enhancing tumor immunity	42
				*In vitro*: Bone marrow derived precursor B cells		
DC-Y13-27	NA	Synthetic compound	Tumor	*In vivo*: Ythdf2-specific knockout mice and subcutaneous tumor-bearing mice	Enhancing the therapeutic effect of radiotherapy, thereby suppressing the NF-κB pathway of MDSCs and enhancing tumor immunity	29
				*In vitro*: Myeloid-derived suppressor cells		

### Activators of YTHDF2

Betaine, a widely distributed methyl donor in animals, plants, and microorganisms, improves cognitive dysfunction in Alzheimer’s disease (AD). It has also been approved by the U.S. Food and Drug Administration (FDA) for the treatment of homocysteinuria, a rare inherited metabolic disorder. It up-regulates YTHDF2 expression in homocysteine (Hcy)-induced rat brains, thus promoting the YTHDF2 recognition of m^6^A sites on the NLRP3 mRNA. This reduces NLRP3 stability, inhibiting the NLRP3/caspase-1/GSDMD pathway and alleviating microglial pyroptosis, thereby ameliorating Hcy-induced cognitive impairment in rats.^80^ However, the current data are limited to rat models, lacking human pharmacokinetic (PK) and long-term toxicity data. To this end, its efficiency across the blood–brain barrier and the clinical dose–effect relationship remain unclear.

Melatonin, an indole heterocyclic hormone secreted by the pineal gland, counteracts inflammation and aging through m^6^A-mediated epigenetic regulation. It is classified as a dietary supplement rather than a drug in the United States; hence, it does not need to be reviewed by the FDA. Melatonin up-regulates YTHDF2 to degrade MAP2K4 mRNA, thereby suppressing the NF-κB pathway and mitigating ovarian aging.^109^ However, melatonin, as a multi-target hormone, may interfere with endocrine homeostasis. Further, the issue of its tissue-specific delivery remains unsolved.

Epigallocatechin gallate (EGCG), the most potent bioactive compound in tea polyphenols, exhibits anti-inflammatory, antioxidant, and antitumor properties. EGCG is also classified as a dietary supplement rather than a drug in the United States and thus does not require review by the FDA. EGCG can help to mitigate obesity by up-regulating YTHDF2, which destabilizes the mRNAs of the adipogenic regulators CCNA2 and CDK2.^150^ This suppresses their protein translation during mitotic clonal expansion, thereby inhibiting early-stage adipogenesis.^150^ However, EGCG exhibits low oral bioavailability (<5%), and high doses can cause hepatotoxicity. Its efficacy remains inadequately validated in non-metabolic inflammatory diseases, such as arthritis. Encapsulation within nanoparticles may offer a promising strategy to improve its stability, efficacy, and pharmacokinetic profile.^151^

In addition to natural compounds, synthetic molecules such as oxaliplatin (OXA) also act as YTHDF2 agonists. OXA was approved by the FDA in 2002 and is mainly used for the treatment of colorectal cancer. OXA activates the cGAS-STING pathway to up-regulate YTHDF2, which uniquely stabilizes Cx3cl1 chemokine transcripts via m^6^A modification, in contrast with YTHDF2’s typical mRNA degradation function. This stabilization recruits CD8^+^ T cells, enhances antitumor immunity, and suppresses hepatocellular carcinoma progression.^45^ Currently, OXA use is limited by its systemic toxicity and challenging application for long-term chronic inflammation; if future work can address these limitations, OXA may be considered a promising therapeutic approach by targeting YTHDF2.

### Inhibitors of YTHDF2

Recent research has identified the synthetic compounds CCI-38 and DC-Y13-27 as potent YTHDF2 inhibitors. However, these two compounds are currently only in the experimental stage and have not yet received FDA approval. Aberrantly high YTHDF2 expression in B-cell acute lymphoblastic leukemia (B-ALL) promotes cancer cell survival and immune evasion through dual mechanisms.^42^ First, YTHDF2 stabilizes m^5^C-modified F-type ATP synthase subunit mRNAs, enhancing ATP synthesis efficiency to fuel rapid tumor growth. Second, it recognizes m^6^A-modified CD19 and MHC class II molecule mRNAs, accelerating their degradation to reduce cancer cell immunogenicity and evade immunotherapy. CCI-38 effectively suppresses YTHDF2 expression, counteracting both oncogenic mechanisms to inhibit B-ALL progression.^42^ CCI-38 is currently only applicable to hematological malignancies and has not yet been tested for effectiveness in solid tumors or classic inflammatory models such as hepatitis and nephritis.

Furthermore, DC-Y13-27 effectively inhibits YTHDF2 expression, demonstrating therapeutic potential in tumors and psoriasis. In ionizing radiation-induced MDSCs, the expression of YTHDF2 is elevated, which activates the NF-κB pathway by degrading the mRNAs of its negative regulators (Adrb2, Mettl, Smpdl3b), promoting MDSC migration and suppressing antitumor immunity.^29^ However, DC-Y13-27 can counteract this by inhibiting YTHDF2, thereby enhancing radiotherapy efficacy, suppressing NF-κB activation in MDSCs, and attenuating immunosuppression to impede tumor progression.^29^ In psoriasis models, DC-Y13-27 stabilizes DKK3 mRNA through YTHDF2 inhibition, blocking the Wnt pathway to reduce inflammation and epidermal hyperplasia and effectively halting disease progression.^141^ However, DC-Y13-27 has a short *in vivo* half-life (< 2 h) and is easily degraded by serum nucleases; additionally, its systemic administration may cause off-target effects in the liver or kidney, which limits its current applicability.^152^

Both natural compounds and synthetic inhibitors targeting YTHDF2 show promising therapeutic potential for inflammatory diseases; however, many still face considerable limitations that require further investigation to ensure their efficacy and safety. Systemic administration of YTHDF2 inhibitors may lead to off-target effects, highlighting the need for tissue-specific delivery systems—such as liposome-encapsulated inhibitors—to enhance localization to affected sites. Developing tissue-specific nanocarriers, exploring conditional knockout strategies, and employing combination therapies will be essential to optimize treatment outcomes and minimize off-target toxicity, ultimately paving the way for more effective clinical treatment strategies.

## Conclusion and perspectives

This article systematically reports the central roles of the m^6^A reader protein YTHDF2 in modulating the inflammatory microenvironment via epitranscriptomic mechanisms. YTHDF2 dynamically regulates macrophage polarization, neutrophil recruitment, NK cell cytotoxicity, and T/B-cell function by degrading or stabilizing key transcripts and pathway components involved in inflammation, thereby influencing tumor immune evasion, the progression of inflammatory diseases, and treatment responses. Furthermore, the review integrates recent advances in agonists and inhibitors targeting YTHDF2—such as DC-Y13-27 and OXA—and proposes novel intervention strategies aimed at reshaping the inflammatory microenvironment by disrupting YTHDF2–RNA interactions. These insights offer new epigenetic avenues to enhance existing treatments, including immune checkpoint therapy.

YTHDF2 exhibits dual cell type-dependent roles in tumor immunity. Most studies indicate that high YTHDF2 expression promotes immune escape by inhibiting chemokine secretion, enhancing tumor glycolysis, and downregulating PD-L1.^58^, ^59^, ^60^ However, in NK cells, its low expression impairs cytotoxic function.^51^^,^^52^ The role of YTHDF2 in CD8^+^ T cells is more complex: high expression inhibits T-cell recruitment, while low expression disrupts cellular homeostasis—both ultimately leading to impaired immune function.^44^^,^^46^^,^^71^ This functional duality highlights its context-dependent behavior, influenced by the specific microenvironment and cellular background. Given the bidirectional regulatory nature of YTHDF2, therapeutic strategies must account for cell specificity. In solid tumors, YTHDF2 inhibitors such as CCI-38 can attenuate the function of immunosuppressive cells. Furthermore, maintaining YTHDF2 activity in NK cells through cytokine stimulation may enhance their antitumor efficacy. Loss of YTHDF2 promotes Th9 cell differentiation and enhances the antitumor activity of CAR-Th9 cells,^153^ offering a promising avenue for adoptive cell therapy. Additionally, combining PD-1 inhibitors may help reverse CD8^+^ T-cell dysfunction, although careful balancing across different immune cell subsets remains essential.

In non-neoplastic inflammatory diseases, YTHDF2 similarly exhibits a dual role. High YTHDF2 expression can promote M1 macrophage polarization and enhance inflammatory responses,^56^^,^^74^ whereas in conditions such as IBD and systemic lupus erythematosus, low expression levels exacerbate the release of inflammatory factors.^65^^,^^69^ It also exhibits different effects on various pathways: its low expression in pathways such as NF-κB/MAPK promotes inflammation, while high expression in the NLRP3/PI3K-AKT pathway promotes inflammation.^78^^,^^106^^,^^121^^,^^132^ Consequently, treatment strategies for inflammatory diseases should be tailored based on the specific target pathway and disease context. For example, YTHDF2 agonists may be beneficial in IBD to counteract excessive inflammation, whereas inhibitors could be more appropriate in NLRP3-driven conditions. Emerging nanodelivery systems, such as pH-responsive lipid nanoparticles (Lip@si-YTHDF2), offer promising approaches for tissue-specific targeting and reduced systemic side effects.^152^ Nonetheless, careful monitoring of immune cell dynamics remains essential for preventing excessive immune activation.

With the expanding use of neoadjuvant immunotherapy to treat both solid tumors^154^ and hematologic malignancies,^155^ the development of novel adjuvant targets to improve immunotherapy efficacy has become a major research focus. As a key epigenetic regulator of the inflammatory microenvironment, understanding the upstream regulatory mechanisms of YTHDF2 is critical for the development of targeted therapeutics. The E3 ubiquitin ligase TRIM8 promotes the ubiquitination and degradation of YTHDF2 via its RING domain,^142^ while the histone acetyltransferase KAT1 activates YTHDF2 transcription by facilitating histone acetylation within its promoter region.^144^ These findings offer potential therapeutic avenues for modulating YTHDF2 expression.

Regarding pharmacological inhibition, two small-molecule inhibitors—CCI-38^42^ and DC-Y13-27^29^—have demonstrated notable potential. These compounds suppress YTHDF2 protein expression and directly interact with its RNA-binding pocket, thereby preventing its association with m^6^A- or m^5^C-modified RNAs and disrupting its downstream regulatory networks. Furthermore, emerging technologies such as PROTACs, nanoparticle-based delivery systems, and CRISPR/Cas9 gene editing offer innovative strategies to achieve targeted degradation or stabilization of YTHDF2, paving the way for interventions with improved specificity and tissue targeting.

The function of YTHDF2 within the inflammatory microenvironment is highly context-dependent, often exerting opposing roles across different immune cells and signaling pathways. Therefore, future research should prioritize the development of precise, cell- or pathway-specific regulatory strategies—such as conditional knockout models, cell type-specific nanodelivery systems, or bispecific small-molecule drugs—to minimize potential off-target effects associated with systemic interventions. Concurrently, it is crucial to continue investigating the expression patterns of YTHDF2 across different immunotherapy patient populations and their correlation with treatment responses to establish predictive biomarkers and advance personalized therapeutic strategies. Ultimately, integrating YTHDF2-targeted therapies with existing modalities such as immune checkpoint inhibitors and cell-based treatments may offer a promising avenue for improving clinical outcomes in patients with refractory tumors.

## CRediT authorship contribution statement

**Junxiu Liu:** Writing – review & editing, Writing – original draft, Visualization, Validation, Supervision, Investigation, Formal analysis, Data curation, Conceptualization. **Senxu Lu:** Writing – review & editing, Visualization, Validation, Investigation. **Chuanhuai Chen:** Validation, Investigation. **Xiaobo Lin:** Validation, Investigation. **Lijuan Xia:** Validation, Investigation. **Pansheng Xu:** Validation, Investigation. **Jinjin Shao:** Validation, Investigation. **Luxi Yang:** Validation, Investigation. **Wenhai Huang:** Writing – review & editing, Validation. **Lijiang Zhang:** Writing – review & editing, Validation, Conceptualization.

## Funding

We gratefully acknowledge the financial supports from Science and Technology Co-construction Project of National Comprehensive Reform Demonstration Area of Traditional Chinese Medicine (No. GZY-KJS-ZJ-2025-043), Basic Research Project of Hangzhou Medical College (No. KYZD2024002), and the National Natural Science Foundation of China (No. 82172688).

## Conflict of interests

The authors declare that they have no competing interests.
