# Supplementary material for: YTHDF2 in inflammation: Mechanisms and therapeutic strategies
Source: Genes Dis. 2025 Oct 28;13(3):101909. doi: 10.1016/j.gendis.2025.101909 (PMC12886528; doi:10.1016/j.gendis.2025.101909)
Supplement: Multimedia component 1 [file mmc1.docx]

**Method:**

For our literature search, we utilized the NCBI Pubmed database (https://pubmed.ncbi.nlm.nih.gov/). The search keywords, along with the number of articles retrieved and the final number and publication years of the included studies, are presented in the table below.

Table4 Retrieve information

| Key words | The quantity retrieved | The quantity included | Year |
| --- | --- | --- | --- |
| YTHDF2 and macrophage | 66 | 21 | 2020-2025 |
| YTHDF2 and neutrophil | 19 | 4 | 2021-2025 |
| YTHDF2 and NK | 14 | 3 | 2021-2024 |
| YTHDF2 and Treg | 3 | 1 | 2023 |
| T cell and YTHDF2 | 47 | 6 | 2021-2024 |
| YTHDF2 and B cell | 100 | 6 | 2020-2025 |
| YTHDF2 and NF-κB | 43 | 10 | 2019-2025 |
| YTHDF2 and MAPK | 14 | 6 | 2019-2024 |
| YTHDF2 and JAK-STAT | 6 | 2 | 2023、2025 |
| YTHDF2 and NLRP3 | 9 | 7 | 2021-2024 |
| YTHDF2 and Toll | 4 | 2 | 2024 |
| YTHDF2 and PI3K | 26 | 8 | 2021-2025 |
| YTHDF2 and inflammation | 81 | 19 | 2020-2025 |

**Abbreviations：**

AD: Alzheimer's disease

AKT: protein kinase B

ALRs: AIM2-like receptors

BMCs: bone marrow cells

CD: Crohn’s disease

CDS: mRNA coding regions

CLRs: C-type lectin receptors

ConA: concanavalin A

DAMPs: damage-associated molecular patterns

DLBCL: diffuse large B-cell lymphoma

DR: diabetic retinopathy

EGCG: Epigallocatechin gallate

ETBF: enterotoxigenic B. fragilis

GCs: Glucocorticoids

GILZ: glucocorticoid-induced leucine zipper

GR: glucocorticoid receptor

GSDMD: gasdermin D

GVHD: graft-versus-host disease

HCC: hepatocellular carcinoma

Hcy: homocysteine

HSPC: hematopoietic stem/progenitor cell

HUVECs: human umbilical vein endothelial cells

IBD: inflammatory bowel disease

IEB: intestinal epithelial barrier

IPF: idiopathic pulmonary fibrosis

IR: ionizing radiation

IRAK: interleukin-1 receptor-associated kinase

IRF3: interferon regulatory factor 3

JAK: Janus kinases

KCs: Kupffer cells

α-KG: α-ketoglutarate

LTB4: leukotriene B4

m^6^A: N6-methyladenosine

m^5^C: 5-methylcytosine

MAPK: Mitogen-Activated Protein Kinase

MCE: mitotic clonal expansion

MDSCs: myeloid-derived suppressor cells

MTC: METTL3/METTL14/WTAP methyltransferase complex

NAFLD: non-alcoholic fatty liver disease

NETs: neutrophil extracellular traps

NK cells: Natural killer cells

NLRs: NOD-like receptors

NLRP3: Nod-Like Receptor Protein 3

OA: osteoarthritis

OIS: oncogene-induced senescence

OSCC: oral squamous cell carcinoma

OXA: oxaliplatin

PAMPs: pathogen-associated molecular patterns

PCa: prostate cancer

PI3K: phosphatidylinositol 3-kinase

PMA: Phorbol 12-myristate 13-acetate

PRR: pattern recognition receptor

RLRs: RIG-I like receptors

RMEC: retinal microvascular endothelial cell

ROS: reactive oxygen species

SASP: senescence-associated secretory phenotype

SIC: sepsis-induced cardiomyopathy

SLE: systemic lupus erythematosus

STAT: signal transducers and activators of transcription

TAMs: tumor-associated macrophages

TANs: tumor-associated neutrophils

TLRs: Toll-like receptors

tMCAO: middle cerebral artery occlusion

TME: tumor mircroenvironment

Tregs: regulatory T cells

UC: ulcerative colitis

YSMPs: yolk sac-derived myeloid progenitors
